# Family History as a Predictor for Disease Risk in Healthy Individuals: A Cross-Sectional Study in Slovenia

**DOI:** 10.1371/journal.pone.0080333

**Published:** 2013-11-06

**Authors:** Zalika Klemenc-Ketis, Borut Peterlin

**Affiliations:** 1 Department of Family Medicine, Medical School, University of Ljubljana, Ljubljana, Slovenia; 2 Department of Family Medicine, Medical School, University of Maribor, Maribor, Slovenia; 3 Clinical Institute of Medical Genetics, University Medical Centre, Ljubljana, Ljubljana, Slovenia; Baylor College of Medicine, United States of America

## Abstract

**Background:**

Family history can be used as a genetic risk predictor for common non-communicable diseases. The aim of this study was to determine the prevalence of healthy individuals at risk of developing these diseases, based on their self-reported family history.

**Methods and Findings:**

This was a cross-sectional observational study. Data were collected in the three largest occupational practices in primary health care centres in Slovenia, a Central European country. The study population consisted of consecutive individuals who came to occupational practices for their regular preventive check-up from November 2010 to June 2012. We included 1,696 individuals. Data were collected by a self-developed questionnaire. The main outcome was the number of participants at a moderate or high risk for the development of cardiovascular diseases, diabetes, and cancer.

The final sample consisted of 1,340 respondents. Moderate or high risk for the development of cardiovascular diseases was present in 280 (20.9%) participants, for the development of diabetes in 154 (11.5%) participants and for cancer in 163 (12.1%) participants.

**Conclusions:**

In this study, we found a significant proportion of healthy individuals with an increased genetic risk for common non-communicable diseases; consequently further genetic and clinical evaluation and preventive measures should be offered.

## Introduction

For centuries, family history has represented an inevitable part of consultation with patients. It enables family doctors to gain insight into the patients’ social background and helps to provide a context for their symptoms, both in terms of possible environmental and lifestyle causes of disease and their concerns about the nature of their illness [1]. Teaching family history taking is also an important part of family medicine education [2]. 

Traditionally, family history taking is focused on patients with symptoms or problems which might imply that the patient has an increased risk of certain chronic diseases, such as diabetes, coronary disease, asthma etc. [3,4]. Rarely, healthy patients or patients with acute problems are asked about their family history [5]. As previous studies have shown, inadequate knowledge of family history in the medically underserved population is a major obstacle, limiting quality management of such patients and often leading to delayed treatment [6].

In recent years, family history has not only been used as a method of social assessment, but also as a genetic risk predictor [7,8]. Specifically, a family history of a particular disease usually reflects the combined effects of genetic susceptibility, shared environmental exposure and common behaviours in relatives [4]. Also, it is known that a family history of a common chronic disease is associated with a 2- to 5-fold relative risk of developing the condition, and this increases with the number of affected relatives [9].

Cardiovascular diseases, diabetes and cancer are the leading causes of the burden of disease and also the leading causes of death from non-communicable diseases in the developed world [10]. Since there is a lot of evidence on the preventable nature of these diseases [11], it is of the utmost importance to detect people at risk at the earliest possible time. One of the possibilities for such early detection is family history. 

A positive family history is a risk factor for the development of cardiovascular diseases, diabetes and cancer. Furthermore, family history also seems to be an independent risk factor for diabetes and cardiovascular diseases [12]. Studies have shown that the presence of a cardiovascular disease in at least one parent doubled the eight-year risk of cardiovascular disease among men and increased the risk among women by 70% [13]. The risk of cardiovascular disease (OR) associated with sibling cardiovascular disease was 2.0, and with parental cardiovascular disease 1.5 [14,15]. Studies in diabetes showed that people with one or more first degree relatives who are affected by diabetes are 2-6 times more likely to have the disease compared with people who have no affected relatives [16]. The risk of diabetes was also highly associated with maternal diabetes (OR = 3.4), parental diabetes (OR = 3.5) and with both parents with diabetes (OR = 6.1) [17]. Studies which focused on the most common cancer sites reported that 5-10% of women with breast cancer had a mother or sister who also contracted the disease, and up to 20% had a first or second degree relative with breast cancer [18]. Also, 5% of adult respondents reported having one or more first degree relatives with colorectal cancer [19].

The Centre for Disease Control (CDC) developed a structured approach for risk assessment of various diseases based on the morbidity and mortality data from a three generation family history [20], which can be used for determining the risk of diseases solely on the data from the family history.

In Slovenia, but also in Europe, there is a lack of data on the prevalence of healthy individuals in the general population at risk of developing cardiovascular diseases, cancer, and diabetes, based on their family history; this study aims to address this gap. 

## Materials and Methods

### Study design and settings

This cross-sectional observational study took place in Slovenia, a central European country with approximately 2,000,000 inhabitants. The national healthcare system in Slovenia can be described as a combination of the Beveridge and Bismarck models; the Bismarck insurance model of financing healthcare is used, but for political reasons there is only one insurance company in Slovenia – the National Health Insurance Institute (NHII). Every inhabitant of Slovenia is insured through their employment status, or, if unemployed, through local communities. Compulsory health insurance covers over 80% of all healthcare costs, and through the purchase of a voluntary insurance top-up payment, the remaining healthcare costs and additional services provided to the customer above the basic level can be covered. The responsibility of the state is to prepare the network of healthcare institutions, which comprises public primary healthcare centres; private family doctors and dentists; pharmacies; specialist services; and public hospitals [21,22].

Data were collected in three occupational practices in primary health care centres (Maribor, Velenje, and Novo Mesto), which carry out obligatory reviews of workers, i.e. preventive check-ups prior to first employment and regular check-ups for employed people. Each worker should be examined by an occupational specialist working in these practices before first employment and every five years during the employment period. 

### Ethical statement

The study received ethical approval from the National Medical Ethics Committee of the Republic of Slovenia (No. 98/12/10).

### Study population

The study population consisted of consecutive individuals who came to occupational practices for their regular preventive check-up from November 2010 to June 2012. The inclusion criteria were an age of 18 years or more and informed written consent for participation in the study. We included 1,696 individuals in the study.

### Data collection

Data were collected by means of a self-developed questionnaire which was completed by participants themselves, with the help of an occupational nurse. It consisted of demographic questions (sex, age, number of siblings and number of father’s and mother’s siblings) and clinical questions (the presence of any chronic disease in the respondents and in their first, second and third degree relatives). The respondents selected the chronic diseases from a list (cardiovascular diseases, diabetes, and cancer of any kind), and were also given the possibility of adding other diseases not included in the list. 

An occupational nurse addressed the participants in the waiting room. She explained the purpose of the study and the procedure and obtained their written informed consent. Then they were given the questionnaire which they completed and returned to the nurse. 

### Determining the level of risk

The Centre for Disease Control (CDC) [20] proposed the following stages for assessing risk of developing diseases based on family history: average risk (only one second-degree relative (grandparents, aunts, uncles, nephews and nieces) with the disease from one or both sides of family; or no family history of disease), moderate risk (only one first-degree (parents and siblings) and one second-degree relative with the disease; only one first-degree relative with the disease; only two second-degree relatives from same lineage with the disease), and high risk (at least two first-degree relatives with the disease; at least one first-degree and two second-degree relatives with the disease from same lineage.

### Statistical analysis

The data were analysed with the SPSS 19.0 package (SPSS Inc, Chicago, IL). Only descriptive statistics were computed. In the statistical analysis, 356 (21.0%) participants were excluded due to the presence of at least one self-reported chronic disease. By using the number and type of affected relatives, we classified respondents into three familial risk levels according to the algorithm adapted from Scheuner et al [20]. In this context, the term “average” risk is used to indicate a baseline population risk of developing disease with minimal or no familial risk.

## Results

### Demographic characteristics of respondents

The final sample consisted of 1,340 respondents, of which 841 (62.8%) were male (Table 1). The mean age of the sample was 40.3 ±10.1 years. 

**Table 1:** Demographic characteristics of the respondents.

Characteristic	Subcategory	Number of respondents	Percentage of respondents
Sex	Male	841	62.8
	Female	499	37.2
Education	Primary school	143	10.7
	Vocational school	515	38.4
	Secondary school	375	28.0
	University	222	16.6

### The characteristics of respondents’ families

The mean number of respondents’ children was 1.5 ±1.0 (minimum 0, maximum 6). The respondents had a mean of 2.1 ±1.7 siblings (minimum 0, maximum 13). Their fathers had a mean of 3.3 ±2.7 siblings (minimum 0, maximum 16) and their mothers 3.2 ±2.5 siblings (minimum 0, maximum 16).

### Family history of diseases

Cardiovascular diseases were the most prevalent diseases in the respondents’ families (Table 2).

**Table 2:** Presence of diseases in relatives.

Disease	Number (%) of first-degree relatives with disease	Number (%) of second-degree relatives with disease	Number (%) of third-degree relatives with disease	Number (%) of all relatives with disease
Cardiovascular diseases	260 (19.4)	29 (2.2)	0	289 (21.6)
Diabetes	147 (11.0)	59 (4.4)	1 (0.1)	207 (15.5)
Cancer	148 (11.0)	49 (3.7)	1 (0.1)	198 (14.8)

### Disease risk

Moderate or high risk for the development of cardiovascular diseases was present in 280 (20.9%) participants; for the development of diabetes in 154 (11.5%) participants; and for cancer in 163 (12.1%) participants (Table 3).

**Table 3:** Disease risk.

Disease	Number (%) of participants with high disease risk	Number (%) of participants with moderate disease risk
Cardiovascular diseases	38 (2.8)	242 (18.1)
Cancer	11 (0.8)	152 (11.3)
Diabetes	5 (0.4)	149 (11.1)

## Discussion

This study showed that, based on their family history, 20.9% of participants had moderate or high risk for the development of cardiovascular diseases, 11.5% for the development of diabetes and 12.1% for cancer. As many as 3% of participants had a high risk of the development of cardiovascular diseases and were therefore in need of genetic counselling. 

In Slovenia, and also in Europe [23,24], there is a lack of data on the prevalence of a family history of most common chronic diseases, especially in the healthy adult population. In 2011, 4.5% of the general Slovenian population had cancer and 9.1% had diabetes, but no data were collected on cardiovascular diseases [25]. In 2011, cardiovascular diseases were the most common cause of death in Slovenia, and diabetes and cancer were among the commonest ones [26]. These facts point to a large public health problem and many preventive campaigns in Slovenia are directed at early detection of these diseases [27-30]. However, not much attention has been directed to the screening of family history, which has already been shown to be useful in conducting a risk assessment [4,31].

In other countries, especially in the USA, some efforts have already been made to gather data on the healthy population at risk of developing cardiovascular diseases, diabetes and cancer based on their family history. The results showed that approximately 50% of healthy respondents had a first or second degree relative with cardiovascular diseases [32], and that 33% of the participants had a higher than average risk of developing cardiovascular diseases [4,31]. Similarly, 30-50% of healthy screened adults had a family history of diabetes in a first degree relative [33] and 11% of healthy individuals had a higher than average risk of developing diabetes [34]. A large study in USA reported that 34% of healthy individuals were at high or moderate risk of at least one of the cancers [35]. 

In our study, the prevalence of first and second degree relatives with cardiovascular diseases, diabetes, and cancer was similar or slightly lower than described in previous studies [17,18,33–35]. Currently, screenings for cardiovascular diseases [27], breast cancer [28], colorectal cancer [30] and cervical cancer [29] are employed in Slovenia as a part of national preventive programmes. However, these programmes start at a relatively late stage in life. For example, screening for cardiovascular diseases starts at the age of 35 for men and 40 for women, whereas screening for colorectal and breast cancer start at the age of 50. 

Since family history seems to be an independent risk factor for diabetes and cardiovascular diseases [12], and since previous studies have shown that it is possible to identify people at risk only through assessing their family history [12,15,20], it becomes obvious that family history should also become an obligatory part of regular check-ups in younger healthy people. Family doctors should therefore regularly ask their patients, especially rare visitors, about their family history. 

It has been shown that to conduct a risk assessment, ideally a full three-generational pedigree should be taken, asking about parents, aunts, uncles, siblings and grandparents on both sides of the family [4,31]. However, this may require up to 30 minutes of family doctors’ time [36] which is often unacceptable or not feasible [37]. Therefore, several questionnaires and computer programmes or tools have been developed to assist family doctors in this task [4,7,36,38]. The best studied is Family Healthware, a family history screening tool aimed at preventing common chronic diseases [15]. 

In order to employ the full potential that assessing risk by family history can offer, we should develop a public screening programme which would start at a younger age (possibly in childhood) [12]. Electronic tools or paper forms for family history taking should be adopted and designed for the easy collection of the data needed in such screening programmes, and should also contain an evidence-based decision-making system with regard to the findings. Such a tool would reduce the time burden of family doctors and enable regular periodic updates on people’s family history, as it is a dynamic structure that is susceptible to change over time[12]. Also, people should be informed about the criteria for genetic susceptibility and the benefits of reporting their family history to their family doctors.

The known family history of young healthy individuals would enable us to provide individually tailored and personalized advice on healthy life styles and also to plan targeted preventive activities which could save a great deal of money. It has been shown that family history has a better predictive value in very common inherited conditions like coronary artery disease than single nucleotide polymorphism-based methods [39]. On the other hand, there is growing evidence that next generation sequencing may provide clinically relevant information, especially in people with a strong family history of a given disease [40]. People at high risk of the development of different diseases should be referred to clinical geneticists for individual genetic counselling, while people with moderate risk could be managed by their family doctors [20].

The strength of this study is the inclusion of all regions in Slovenia. Slovenia is geographically a very diverse country and we were able to include all the main regions in the sample. However, the sample was not representative of the general population as it only included individuals who were employed (and therefore probably healthier than the overall general population) which could be a source of a selection bias. Another limitation is the problem of self-reporting of data; they were not checked in medical records or through personal interviews with relatives. The results might therefore be subjected to a recall bias. 

## Conclusion

This study is the first study on this subject in Slovenia and one of very few in Europe. It provides us with the first information on the relevance of family history at a time when genetic medicine is becoming more and more important. It also points to the need for genetic counselling by clinical geneticists for the healthy population at high risk. There is a need for further studies, possibly on larger samples collecting objective information on family history.
